# Navigating the nuances: comparative analysis and hyperparameter optimisation of neural architectures on contrast-enhanced MRI for liver and liver tumour segmentation

**DOI:** 10.1038/s41598-024-53528-9

**Published:** 2024-02-12

**Authors:** Felix Quinton, Benoit Presles, Sarah Leclerc, Guillaume Nodari, Olivier Lopez, Olivier Chevallier, Julie Pellegrinelli, Jean-Marc Vrigneaud, Romain Popoff, Fabrice Meriaudeau, Jean-Louis Alberini

**Affiliations:** 1 Institut de Chimie Moléculaire de l’Université de Bourgogne, ICMUB UMR CNRS 6302, Université Bourgogne, 21000 Dijon, France; 2 Service de Médecine Nucléaire, Centre Georges-François Leclerc, 21000 Dijon, France; 3 Service de Radiologie et Imagerie Medicale Diagnostique et Therapeutique, Centre Hospitalier Universitaire, 21000 Dijon, France

**Keywords:** Cancer imaging, Cancer therapy, Cancer, Machine learning, Programming language

## Abstract

In medical imaging, accurate segmentation is crucial to improving diagnosis, treatment, or both. However, navigating the multitude of available architectures for automatic segmentation can be overwhelming, making it challenging to determine the appropriate type of architecture and tune the most crucial parameters during dataset optimisation. To address this problem, we examined and refined seven distinct architectures for segmenting the liver, as well as liver tumours, with a restricted training collection of 60 3D contrast-enhanced magnetic resonance images (CE-MRI) from the ATLAS dataset. Included in these architectures are convolutional neural networks (CNNs), transformers, and hybrid CNN/transformer architectures. Bayesian search techniques were used for hyperparameter tuning to hasten convergence to the optimal parameter mixes while also minimising the number of trained models. It was unexpected that hybrid models, which typically exhibit superior performance on larger datasets, would exhibit comparable performance to CNNs. The optimisation of parameters contributed to better segmentations, resulting in an average increase of 1.7% and 5.0% in liver and tumour segmentation Dice coefficients, respectively. In conclusion, the findings of this study indicate that hybrid CNN/transformer architectures may serve as a practical substitute for CNNs even in small datasets. This underscores the significance of hyperparameter optimisation.

## Introduction

Medical image segmentation is a crucial and extensive research domain, acknowledged in both the computer vision and medical image analysis communities^1^. It plays a critical role throughout the healthcare process, including clinical diagnosis, treatment planning and follow-up^1–3^.

Accurate tumour segmentation is crucial in the context of Selective Internal Radiation Therapy (SIRT). SIRT is a specialised treatment approach commonly utilised for liver tumours, whereby radioactive microspheres are delivered directly into the blood vessels supplying the tumour, precisely targeting it while sparing healthy tissue. In this procedure, an exact segmentation of the liver and the tumour ensures an optimal dosimetry calculation, which leads to the efficacy and safety of the SIRT treatment. Therefore, enhancing segmentation results in more accurate dosimetry^4^, leading to a more efficient treatment approach.

In recent years, deep learning methods have emerged as the primary approach to achieve state-of-the-art results in various medical image segmentation operations, including organ and tumour segmentation^5–7^. This aspect makes them relevant for SIRT treatment planning.

Initially, architectures based on Convolutional Neural Networks (CNNs) designed for classification, including AlexNet^8^, ResNet^9^, VGG^10^, and Inception^11^, were adapted and used as basic units for segmentation. Subsequently, the structured “U-shaped” encoder-decoder model, exemplified by U-Net^12^, was introduced. This method has proven to be an efficient answer to the problems of semantic segmentation, consistently yielding excellent results. However, there has been a recent application of transformer-based structures^13^, originating from natural language processing, to the field of computer vision, resulting in a new group of architectures exhibiting superior performance as compared to CNN architectures in certain tasks^14,15^. Transformer-based architectures have been applied in medical imaging^16–18^. Nonetheless, the superiority of transformers compared to CNNs has not been established yet in small datasets, where CNNs can still outperform transformer-based architectures due to the requirement of a large amount of data to exploit the full capacity of this type of architecture. Thus, the circumstances in which transformers can outperform CNNs are unclear. This proliferation of models complicates the selection of a candidate for a specific application.

**Figure 1:**
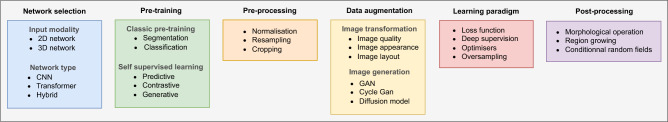
A visual representation of a deep learning pipeline for medical imaging, divided into six key stages: 1) Network Selection, 2) Pre-training, 3) Pre-processing, 4) Data Augmentation, 5) Learning paradigm, and 6) Post-processing.

In addition to architectures, the training strategy can also impact model performance. In this paper, the combinations of the proposed architecture and the proposed training strategy will be referred as a pipeline. Figure 1 illustrates the most common categories and subcategories involved in a tailored deep learning pipeline for medical imaging. Each of these choices can lead to noteworthy improvements in the precision and clinical utility of the final network output.

Now, examining individually each block illustrated in Fig. 1, pre-training a model^8^ enables the model to initialise with weights adjusted to a similar data distribution, potentially leading to faster convergence and improved performance. The sub-field of pre-training known as self-supervised learning^19^ is highly beneficial in medical imaging. Unlike pre-training, it can be implemented on images without annotations^20^.

Handling 3D medical images that potentially exhibit noteworthy differences between images necessitates data preprocessing in order to standardise the dataset. Such preprocessing usually includes procedures such as normalising intensity levels and re-sampling to guarantee that the input data are of homogeneous throughout the entire dataset.^21^.

Data augmentation methods^22–25^ including random rotation, scaling, and flipping can be used to artificially enhance the size of the training dataset and enhance the generalisation capabilities of models.

Learning paradigms are utilised to regulate model progression in training. In particular, supervised loss functions play a crucial role in computing the difference between predictions and labels. Subject to the task and intended results, the choice of loss function varies. The most commonly employed options include cross-entropy-based and dice coefficient-based losses^26,27^. Similarly, the chosen optimiser can greatly affect both the speed of training and the final performance. Its role is to optimise the model weights based on the derivative value of the loss function in order to minimise the value of the function itself. Among various optimisers, stochastic gradient descent (SGD)^28^, Adam^29^, and AdamW^30^ are the most popular choices.

Finally, the segmentation results can be refined and any remaining artefacts or noise can be removed by using post-processing techniques, such as region growing^31^, conditional random fields^32^, or mathematical morphology operations, including dilation, erosion, opening, or keeping the largest connected component.

When deep learning pipelines are published, they are typically optimised and designed for a specific task, which can lead to a decrease in performance when applied to different tasks or datasets. In an effort to address this issue, frameworks like nnUNet^33^ have been introduced to enhance generalisation across heterogeneous datasets. Building on this idea, the objective of this study is to combine and compare the training strategies of seven promising deep learning pipelines for 3D medical image segmentation specifically within the context of SIRT treatment planning, with a focus on liver and tumour segmentation. To this end, this study combines the different elements of the seven pipelines into a single one, and optimises the performance of the seven corresponding architectures by varying and adapting the value of each hyperparameter using Bayesian search. With this approach, the study aims to identify the most effective strategies for 3D liver tumour segmentation. This study focuses on network selection, pre-processing, data augmentation and learning paradigm. All models are trained and optimised on the A Tumor and Liver Automatic Segmentation (ATLAS)^34^ dataset, which consists of 3D contrast-enhanced magnetic resonance images (CE-MRI) of the liver and tumour with annotations for patients presenting hepatocellular carcinoma (HCC).

Several studies using comparable datasets have been documented in the scientific literature but on private datasets. Christ et al.^35^ performs Diffusion-Weighted MRI (DW-MRI) segmentation on 31 patients with HCC using cascaded fully convolutional neural networks, Zhao et al.^36^ proposed liver tumour detection through the using of generative adversarial networks on 131 patient with HCC, and similarly, Kim et al.^37^ performed region-of-interest detection of HCC on a multi-centre CE-MRI dataset containing 545 patients. Xiao et al^38^ introduced a liver tumour segmentation solution employing radiomic features from T2 delay-phase CE-MRI over 200 patients. Zhao et al.^39^ contributed to liver tumour segmentation on multi-modal non-contrast MRI on 255 HCC patients, while Zheng et al.^40^ leveraged multi-phase dynamic 4D CE-MRI for segmentation on a dataset including 190 HCC patients.

This study distinguishes itself from the existing literature by providing a comprehensive and unbiased comparison of the most promising architectures for 3D medical image segmentation. In addition, it explores the impact of different training strategies. In particular, this work goes further than previous publications by examining the impact of each hyperparameter in relation to both the other hyperparameters and the network architecture. This approach provides a more nuanced understanding of the factors that contribute to the performance of deep learning models for medical image segmentation.

The main contribution of this work can be summarised as follows:

- Training strategies optimisation: This work presents a detailed study of the impact of various training strategies on seven advanced architectures for 3D medical image segmentation.
- Comparative analysis using publicly available CE-MRI data: The study offers a unique comparative analysis of liver and tumour segmentation using publicly available CE-MRI data, distinguishing it from prior research.
- In-depth architectural comparison: It provides a comprehensive comparison of CNN, transformer, and hybrid architectures using the ATLAS dataset, contributing to a better understanding of their relative strengths and weaknesses in medical image segmentation.
- Hyperparameter evaluation: The research evaluates critical hyperparameters and contributes to the understanding of their role in optimising segmentation models.
- Resources: A GitHub repository containing code that facilitates the training and tuning of the seven deep learning architectures for 3D segmentation tasks and reproduces the results: gitlab.in2p3.fr/iftim/public-projects/navigating-the-nuances.

These contributions provide valuable insights into the factors that influence the performance of deep learning models in medical image segmentation along with practical tools and recommendations to improve their accuracy and generalisability. The objective of this study is to determine the optimal configuration (architecture and training strategy) on the ATLAS dataset.

## Materials and methods

### Dataset

The ATLAS^34^ dataset was used in this study to design and evaluate the complete optimisation process. The ATLAS dataset was chosen for this study on MRI-based liver tumour segmentation due to the lack of research in this area, unlike CT, where the LiTS^41^ dataset is popular. This choice aims to address the research gap, especially since MRI is widely used in clinical environment. The study also compares transformer and CNN models on smaller datasets, exploring the potential of transformers in a field dominated by CNNs. This decision reflects a strategy to provide new insights in under-researched areas and test various neural network architectures in clinically relevant, scenarios.

**Figure 2:**
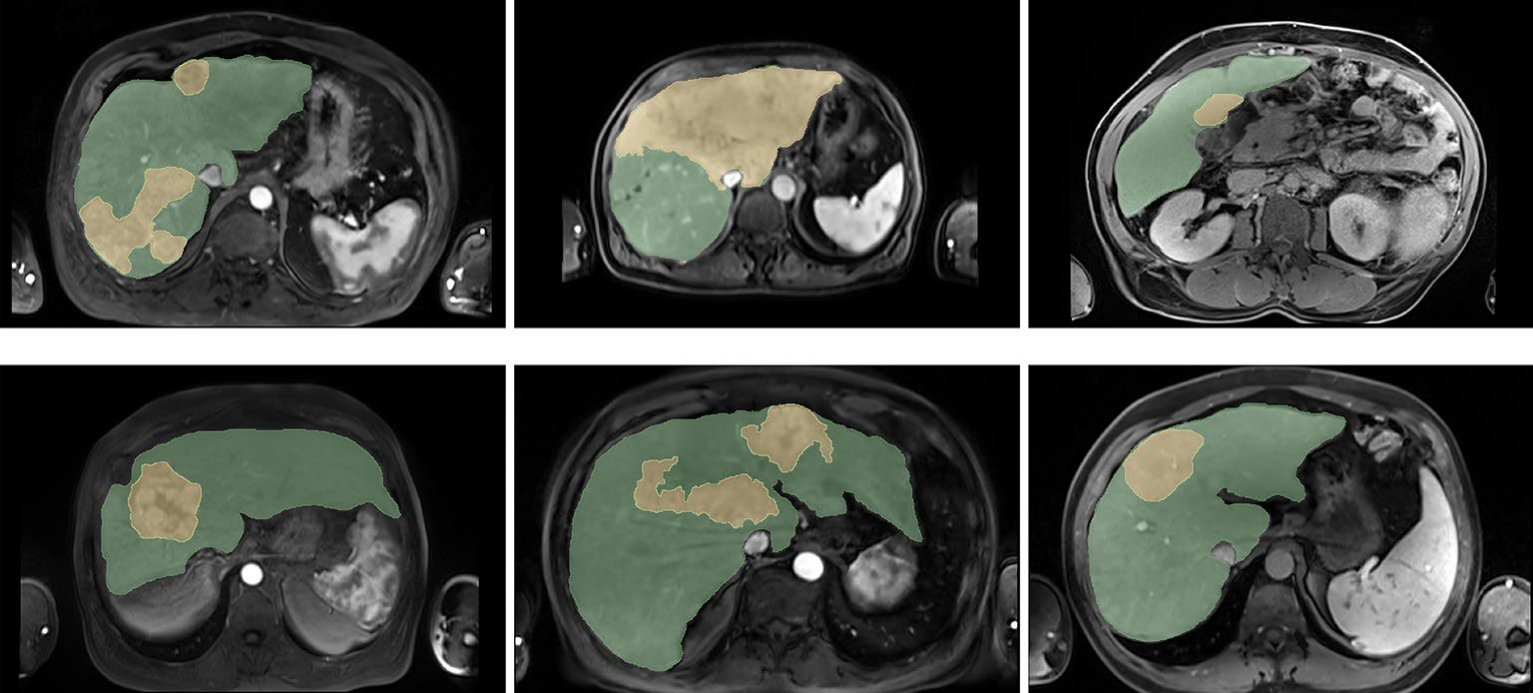
Axial slices of six contrast-enhanced magnetic resonance images from six different patients with the corresponding image labels superimposed. The liver appears in green and tumour in yellow.

The dataset is made up of 90 contrast-enhanced magnetic resonance images (CE-MRI), collected from 90 patients with hepatocellular carcinoma (HCC). Alongside the CE-MRI, label images of the liver and tumours were provided. The labels were manually delineated by an experienced MRI radiologist using the MIM SurePlan LiverY90 software^42^ from the transversal view CE-MRIs. As shown in Fig. 2, there are three classes in this dataset: background, liver and tumour.

The ATLAS dataset contains 3D CE-MRI of the whole liver and tumour, consisting of 44 to 136 transverse slices of the thorax and abdomen. The pixel spacing of each slice in the dataset ranges from ${0.68 \times 0.68}\,\textrm{mm}^{2}$ to $1.41 \times 1.41\, \textrm{mm}^{2}$, with a slice thickness of 2 mm to 4 mm. Bias field correction was applied to every image.

The ATLAS dataset is composed of two distinct sets. The training set which contains 60 images and the testing set with 30 images. To evaluate the performance of each model in this study, we randomly divided the training dataset into two sets: 48 images were used to train the models, and 12 images were reserved to validate them. The test set was used to measure the optimisation process’s impact.

### Model baselines

This work groups seven different deep learning-based pipelines, optimised for seven different deep learning tasks. Each of the pipelines has demonstrated remarkable efficacy for their respective task.

**Figure 3:**
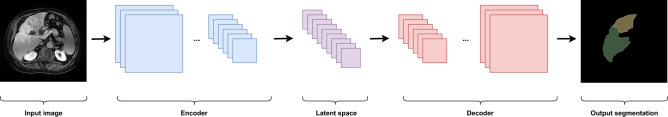
Classic encoder / decoder pattern for image segmentation.

All the seven chosen architectures have been designed using an encoder/decoder scheme illustrated in Fig. 3, to process 3D images for the purpose of medical image segmentation of MR or CT images. These architectures are described in Table 1. Two are CNNs, one is a pure transformer network, and the remaining four are hybrid CNN/transformer architectures.

**Table 1:** Main characteristics of the models studied.

Model	Category	Main characteristics
nnFormer^43^	Hybrid	Local and global attention blocks, convolutionnal down-sampling
nnUNet^33^	CNN	U-Net featuring five encoder and decoder blocks
SegmentationNet^44^	CNN	U-Net featuring four encoder-decoder blocks
Swin-UNetr^45,46^	Hybrid	Swin-Unet^47^ based encoder, CNN decoder, pre-trained with SSL
Transbts^48^	Hybrid	Large transformer bottleneck, CNN encoder/decoder
UNetr^49^	Hybrid	Transformer based encoder, CNN decoder
VT-UNet^50^	Transformer	Alternates regular and shifted attention windows layer, pre-trained with Swin T^14^

To achieve a fair comparison between the seven chosen deep learning architectures, a consistent methodology was introduced. Individual components from every pipeline were extracted and then merged to create a combined pipeline. This procedure enables us to fairly evaluate the impact of each component of the deep learning pipeline on the quality of the segmentation.

### Optimisation strategy

To optimise a model’s performance through hyperparameters tuning, three optimisation strategies can be considered: random search, grid search, and Bayesian search^51,52^. Random search involves randomly sampling hyperparameter values within specified ranges, whereas grid search exhaustively explores all possible combinations of hyperparameters. By contrast, Bayesian search is a hyperparameter optimisation technique that utilises a probabilistic model to improve the new hyperparameter combinations based on previous evaluations. The search is guided by information from earlier trials towards promising areas in the hyperparameter space where optimal configurations are more likely to be located. Through iterative selection of new hyperparameter configurations for evaluation based on the model’s predictions, Bayesian search effectively reduces the search space and identifies the most suitable hyperparameters for a machine learning model. Thus, all the selected architectures were trained multiple times using various hyperparameter configurations, in compliance with the Bayesian search optimisation strategy from WandB^53^, which is based on the scikit-learn implementation^54^.

Due to the considerable number of hyperparameter combinations, the optimisation process was carried out in several consecutive phases and some hyperparameters were fixed. This approach extends Bayesian search, providing a further decrease in the number of combinations and enables for a deeper exploration of the impact of similar hyperparameters relative to one another.

Therefore, the studied hyperparameters were categorised into three groups and optimised during three successive optimisation phases: the patch size optimisation phase, the data pre-processing and data augmentation optimisation phase, and the learning paradigms optimisation phase. During each phase, for each of the seven architectures, the same set of hyperparameters was optimised. The optimised configuration of hyperparameter per architecture is then saved and carried forward into the next optimisation phase.

### Validation metrics

The model’s performance was measured on the validation set at each epoch during training. Due to the incapacity of the models to process large 3D images at once on a 32 GB Graphics Processing Unit (GPU), a sliding window approach was adopted in validation with a $50\%$ overlap. In this study, inspired by the nnUNet pipeline, we utilise an exponential moving average of the Generalised Dice (GD)^55^ score as our validation metric for evaluating segmentation quality. This method is specifically chosen to select models at a phase of their training that consistently exhibit strong generalisation capabilities across the test dataset. The GD metric, adjusted for class imbalance, ensures a balanced assessment between different classes. Using an exponential moving average, we blend both historical and current evaluation of the performances, offering a more comprehensive and stable evaluation of the model’s generalisation ability. This approach lessens the risk of over-fitting on a small validation set, thus providing a more trustworthy indicator of the model’s performance on unseen data. The GD is defined as follow:

1 $$\begin{aligned} \text {GD}(A, B) = \frac{2 \sum _{i=1}^{N} w_i |A_i \cap B_i|}{\sum _{i=1}^{N} w_i (|A_i| + |B_i|)}, \end{aligned}$$

where N is the number of classes (three in our case), *A* and *B* are two sets representing the predictions and labels, respectively. $A_i$ and $B_i$ ($\forall i\in \llbracket 1, N \rrbracket$) represent the predictions and labels for class *i*, respectively and |.| denotes the cardinality or number of elements. The weight $w_i$ ($\forall i\in \llbracket 1, N \rrbracket$) is used to handle class imbalance, and is usually set to the inverse of the square of the number of pixels in each label class $w_i = 1 / |B_i|^{2}$.

With $\text {GD}_{i}$, the generalised dice at epoch *i*, the validation metric (VM) at the same epoch is defined as:

2 $$\begin{aligned}{} & {} \text {VM}_0 = \text {GD}_0 \end{aligned}$$

3 $$\begin{aligned}{} & {} \text {VM}_i = 0.9 \times \text {VM}_{i-1} + 0.1 \times \text {GD}_i \end{aligned}$$

### Evaluation metrics

To quantitatively analyse the quality of the segmentation on the test set, the following metrics were selected:

1. *Dice Coefficient* It measures the similarity between two sets. Given a prediction and a ground truth per pixel for a fixed class the Dice is defined as:
  4 $$\begin{aligned} \text {Dice} = \frac{2 \times \text {TP}}{\text {TP} + \text {FP} + \text {FN}}, \end{aligned}$$
  where TP, FP and FN respectively corresponds to the number true positives, false positives and false negatives.
2. *5mm Surface Dice* This metric provides a variant of the Dice coefficient which is calculated within a 5mm distance from the surfaces of the structures:
  5 $$\begin{aligned} \text {SD}_{5\text {mm}} = \frac{2 \times \text {TP}_5}{\text {TP}_5 + \text {FP}_5 + \text {FN}_5}, \end{aligned}$$
  where $\text {TP}_5$, $\text {FP}_5$ and $\text {FN}_5$ correspond to the number of true positives, false positives, and false negatives subsets of pixels located within 5mm of the surfaces from the structure studied.
3. *Precision* Also known as the positive predictive value, it quantifies the accuracy of positive predictions. Precision can be defined:
  6 $$\begin{aligned} \text {Precision} = \frac{\text {TP}}{\text {TP} + \text {FP}}, \end{aligned}$$
  where TP and FP respectively corresponds to the number true positives, false positives.
4. *Recall* Also known as sensitivity or true positive rate, it quantifies the ability to detect positive instances. Recall is defined as:
  7 $$\begin{aligned} \text {Recall} = \frac{\text {TP}}{\text {TP} + \text {FN}}, \end{aligned}$$
  where TP and FN respectively corresponds to the number true positives, false negatives.
5. *Hausdorff Distance (HD)* It represents the greatest of all the distances from a point in one set to the closest point in the other set. Mathematically, for two-point sets $A$ and $B$:
  8 $$\begin{aligned} \text {HD}(A, B) = \max \left( \max _{a \in A} \min _{b \in B} d(a, b), \max _{b \in B} \min _{a \in A} d(a, b) \right) , \end{aligned}$$
  where $d(a, b)$ is the Euclidean distance between points $a$ and $b$.

To assess the significance of each metric, a Shapiro-Wilk test was first conducted on the paired differences between the results before and after optimisation. Based on the results of this test, either a paired t-test or a Wilcoxon signed-rank test was then applied to each metric to determine its statistical significance.

### Experiments

#### Baseline

Prior to any optimisation, each architecture was trained as described in the published articles. These baseline results are used as a starting point to measure the impact of the optimisation process.

#### Patch size influence

If studies indicate that a larger patch size leads to improved performance^56–59^, this gain may be negligible and would result in a significant increase in training time. Therefore, using intermediate patch sizes could potentially be more efficient. To this end, three patch size configurations (small, intermediate, and large) were tested for each architecture.

The largest patch size compatible with a 32GB GPU and a batch size of two was selected. For each patch size, the depth was fixed at 64, which covers a substantial portion of the images along that axis. This is because the images have a median size of 80 pixels in that dimension. Width and height patch sizes vary in different configurations. The intermediate patch size is one-fourth the size of the largest patch size, and the small patch size is 16 times smaller than the largest configuration. Consequently, the overall patch size ranges from $64 \times 64 \times 64$ to $384 \times 304 \times 64$, depending on the architecture.

Table 2 gives the detailed patch sizes used in this study. The values in italic represent the baselines patch sizes that are the closest to those proposed by the authors of the seven pipelines on similar datasets. Since only three patch size combinations were tested, Bayesian search was not applied during this phase.

**Table 2:** Details of the different patch sizes tested in this study. Italic correspond to baseline configurations.

Hyperparameter	Small	Intermediate	Large
nnFormer patch size	$64 \times 64 \times 64$	$\textit{128} \times \textit{128} \times \textit{64}$	$224 \times 224 \times 64$
nnUNet patch size	$96 \times 96 \times 64$	$192 \times 160 \times 64$	$\textit{384} \times \textit{288} \times \textit{64}$
Swin-UNetr patch size	$64 \times 64 \times 64$	$128 \times 128 \times 64$	$\textit{256} \times \textit{224} \times \textit{64}$
Transbts patch size	$64 \times 64 \times 64$	$\textit{128} \times \textit{128} \times \textit{64}$	$224 \times 224 \times 64$
UNetr patch size	$96 \times 96 \times 64$	$\textit{192} \times \textit{160} \times \textit{64}$	$384 \times 304 \times 64$
UNet3d patch size	$\textit{96} \times \textit{96} \times \textit{64}$	$192 \times 160 \times 64$	$384 \times 304 \times 64$
VT-UNet patch size	$64 \times 64 \times 64$	$\textit{128} \times \textit{128} \times \textit{64}$	$256 \times 224 \times 64$

#### Data pre-processing and data augmentation influence

Data pre-processing and data augmentation are among the most important aspects of the learning process in deep learning algorithms, as they provide robustness to the models^21–25^.

The main objective of data pre-processing is to standardise a dataset^21^. Therefore, a range of pre-processing techniques could be employed such as image resampling, image cropping, and image normalisation. The large part of the studied pipelines uses median resampling since it appears as a fair compromise between image quality and memory consumption. Thus, all images were resampled to the median spacing of the dataset: 1.04 $\times$ 1.04 $\times$ 3.00 mm^3^ leading to images after resampling between $320 \times 250 \times 44$ pixels ($320 \times 250 \times 132$ mm) to $512 \times 512 \times 136$ pixels ($512 \times 512 \times 408$ mm). The ATLAS dataset presents a significant class imbalance among the different structures (background $>>$ liver $>>$ tumour). To address this, an oversampling strategy^60^ was also fixed for every training iteration.

During the optimisation process, the only studied hyperparameter that affected the pre-processing was the image normalisation strategy. Pixel intensities vary significantly across different images due to the various machines and sequences used to acquire the ATLAS dataset. Consequently, inter-image normalisation strategies were not taken into consideration. However, two intra-normalisation strategies, namely, min–max normalisation and Z-score normalisation, were analysed.

Min–max normalisation consists of re-scaling the pixel intensities of an image between 0 and 1 and is defined as:

9 $$\begin{aligned} x' = \frac{x - \min (X)}{\max (X) - \min (X)}, \end{aligned}$$

where *x* is the original pixel intensity value, $x'$ is the normalised pixel intensity value, and *X* the ensemble of pixel values in the image.

Z-score normalisation, is another widely-used technique to re-scale the pixel intensities of images. Z-score normalisation involves re-scaling the pixel intensities to have zero mean and unit variance and is defined as:

10 $$\begin{aligned} x' = \frac{x - \mu (X)}{\sigma (X)}, \end{aligned}$$

where *x* is the original pixel intensity value, $x'$ is the normalised pixel intensity value, $\mu (X)$ is the mean pixel intensity value in the image, and $\sigma (X)$ is the standard deviation of pixel intensity values in the image.

Data augmentation enables the artificial expansion of a training dataset, resulting in more resilient algorithms with superior generalisation capabilities. This is particularly crucial when working with medical image datasets, which usually have limited data. Data augmentation can be categorised into two forms, image transformation augmentation^23^ and image generation-based augmentation^61^. In this study, unlike^62^, only image transformation strategies were considered.

Image transformation-based augmentation involves applying a set of transformation to the images. Transformations such as image flipping, image rotation, intensity scaling, and intensity shifting are widely accepted in the literature^62^ and are already utilised in most of the seven original pipelines. Therefore, they were systematically applied.

In addition to these standard transformations, six other data augmentation transformations were implemented in the seven selected pipelines. To simplify the optimisation process, we divided this augmentation techniques into three distinct groups: the image fidelity group (IF) with Gaussian noise and Gaussian blur, the scaling and resolution group (SR) with zoom and low image quality simulation and the luminance and contrast group (LC) with contrast and gamma (inverted and non-inverted) alterations.

Each group consists of two closely related augmentation methods, that help to reduce the combinatorial complexity. This leads to three hyperparameters to tune during the optimisation process. The detailed parameterisation for each augmentation method is provided in Table 3.

**Table 3:** Detailed parameterisation per augmentation method.

Data augmentation	Probability	Range
**Fixed hyperparameters**		
Rotations	0.1	0 – 45^∘^
Flipping	0.1	_
Intensity shifting	0.1	0 – 0.1
Intensity scaling	0.1	0 – 0.1
**Optimised hyperparameters**		
Gaussian noise	0.1	_
Gaussian blur	0.1	_
Zoom	0.1	0.7 – 1.4
Low image quality simulations	0.5	0.5 – 1.0
Contrast	0.1	0.75 – 1.25
Gamma non-inverted images	0.3	0.7 – 1.5
Gamma-inverted images	0.1	0.7 – 1.5

The selected values were based on the values used in the selected pipelines.

Thus, the impact of four hyperparameters was considered for this optimisation phase (normalisation, image fidelity, scaling and resolution, luminance and contrast). The normalisation parameter being either min–max or z-score and each data augmentation hyperparameter was either on or off, it leads to 16 possible combinations. Eight of these combinations were tested for each architecture using a Bayesian search optimisation strategy. The best hyperparameter configuration identified during the patch size optimisation phase was used for comparison.

#### Learning paradigms influence

Learning paradigms control a model’s progression during the training. In supervised segmentation learning, a loss function quantifies the difference between the model’s prediction and a label. Loss functions for medical image segmentation can be categorised into four categories: distribution-based, region-based, boundary-based (they are not covered in this paper), and compound-based^27^.

**Distribution-based losses:** the objective of distribution-based loss functions is to reduce the differences between two distributions, namely the predicted and target distributions. The cross-entropy (CE) loss based on the Kullback-Leibler divergence serves as the foundation for other functions in this category. CE loss can be defined as:

11 $$\begin{aligned} \text {CE Loss} = -\frac{1}{I} \sum \limits _{j=1}^{J} \sum \limits _{i=1}^{I} y_{ij} \log (\hat{y}_{ij}), \end{aligned}$$

where *I* is the number of voxels, *J* is the number of classes, $y_{ij}$ is the binary indicator for the class *j* according to the ground truth and $\hat{y_{ij}}$ the probability of the pixel *i* to belong to the class *j* according to the prediction.

**Region-based losses:** region-based losses minimise discrepancies between predicted segmentation and ground truth by optimising overlap. Functions of this category are derived from the Dice loss function, which involves optimising the Dice Similarity Coefficient. This metric is commonly used to evaluate medical image segmentation tasks. The Dice loss is defined as:

12 $$\begin{aligned} \text {Dice Loss} = 1 - \frac{2}{J}\sum \limits _{j=1}^{J} \frac{\sum \limits _{i=1}^{I} y_{ij} \hat{y}_{ij} + \epsilon }{\sum \limits _{i=1}^{I} y_{ij} + \sum \limits _{i=1}^{I} \hat{y}_{ij} + \epsilon }, \end{aligned}$$

where *J* represents the number of classes, *I* the number of pixels in the images, and $\hat{y}_{ij}$ and $y_{ij}$ represent the predicted probability and the ground truth label for each pixel *i* in the image to belong to class *j*. The $\epsilon$ term is added for numerical stability and is usually set to a small positive value (e.g., $1e-5$). If a second version of the Dice loss exists with square terms at the denominator, this version will not be discussed here according to the results of Ma et al.^27^.

**Compound losses:** compound losses combine previous losses, including losses from different categories. The DiceCE loss^33^ is defined as the addition of the CE loss and the Dice loss. The joint use of Dice loss and cross-entropy loss can improve segmentation performance by taking advantage of each loss function^27^. Combining both loss functions allows to consider both local accuracy and global consistency in segmentation, maximising segmentation similarity while minimising class probability distribution. The DiceCE loss is defined as follows:

13 $$\begin{aligned} \text {DiceCE Loss} = 0.5 \times \text {CE Loss} + 0.5 \times \text {Dice Loss} \end{aligned}$$

Although the literature presents numerous loss functions across these three categories, this study will exclusively focus on the two loss functions, Dice and DiceCE, as they are the only two loss functions utilised by the seven pipelines analysed.

Choosing a loss function includes deciding whether to include or not the background class in the loss calculation. Consequently, the effect of the background on performance was also investigated.

While a loss function allows the measurement of the difference between ground truth and prediction, the optimiser controls how the model evolves with respect to that difference. The seven pipelines deploy three distinct optimisers: the Stochastic Gradient Descent (SGD) with Nesterov momentum^28^, the Adaptive Moment Estimation (Adam)^29^ and AdamW^30^.

The SGD optimiser computes the gradients for each parameter and modifies the parameters of the model based on a fraction of the gradient dependent on the value of the learning rate. Utilising a momentum term to consider the preceding gradient enhances convergence and retains momentum in a particular direction. The Nesterov version of the momentum algorithm is an enhancement to the basic momentum update as it computes the gradient after the momentum update, resulting in a more precise direction towards the minimum of the loss function.

Adam combines the concepts of momentum and adaptive learning rates in order to maintain two moving averages for every parameter and adjust the learning rates for each parameter as training progresses. The utilisation of these moving averages aids in stabilising the updates and countering the problems of vanishing or exploding gradients, leading to faster and more stable convergence.

AdamW builds upon Adam by introducing a weight decay regularisation term. This term aids in preventing overfitting by applying a penalty to the magnitude of the weights, steering the model towards simpler, more general representations. AdamW decouples the weight decay from the adaptive learning rate updates, allowing the model to use the benefits of Adam while also employing weight decay for improved generalisation.

The impact of the three optimisers is studied in this phase, and their configurations are detailed in Table 4.

**Table 4:** Optimiser configuration.

Optimiser	Learning rate	Regression weight
Adam	$1e^{-4}$	$3e^{-5}$
AdamW	$1e^{-4}$	$1e^{-2}$
SGD	$1e^{-2}$	$3e^{-5}$

Overall, the three parameters (two losses, inclusion or not of the background class, and three optimisers) lead to 12 possible hyperparameter configurations. During this optimisation phase, eight different combinations per model were tested using Bayesian search. The optimal hyperparameter configuration identified during the data pre-processing and data augmentation optimisation phase was used as point of comparison.

Once the optimisation process completed through the three optimisation phases, the best configuration for each model according to the value of the validation metric is evaluated on the test set and compared to the initial baseline configuration.

#### Implementation Details

All experiments described in this document were conducted using the PyTorch^63^ library (version 1.11.0) along with the NVIDIA Compute Unified Device Architecture (CUDA) toolkit^64^ (version 11.3.1). The training and inference of the different architectures were performed using Tesla V100S-PCIE-32GB GPUs. Data processing was performed using the Medical Open Network for Artificial Intelligence (MONAI)^65^ frameworks (version 0.8.1).

Regarding the hyperparameters not mentioned so far, the models were trained in their original configuration optimised by the authors for their own datasets. No post-processing was applied.

## Results

### Baseline

When examining the baseline inter-model performance outlined in Table 5, a significant discrepancy is observed. There is a considerable gap in performance as the validation metric value ranges from 39.4 points for UNETR to 69.0 points for nnUNet. Whilst UNETR appears to be a performance outlier, there remains a difference of 19.4 points between nnUNet and Swin-UNETR, the second lowest performing model.

**Figure 4:**
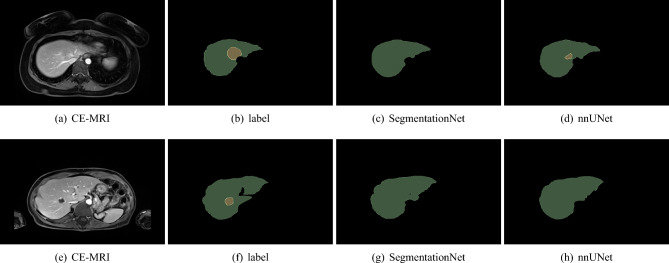
Complex tumour segmentation cases with SegmentationNet and nnUNet. The liver appears in green, and the tumour in yellow.

Figure 4 highlights that the majority of the models struggle to accurately identify the tumour’s location in the most complex cases. In one of the two images, nnUNet is able to classify a few pixels as tumour, but mid-range models such as SegmentationNet are unable to correctly classify any pixels as tumour.

### Patch size influence

The effect of the influence of the patch size on the validation set is detailed in Table 5. Online Appendices Tables S1 and S2 provide the test set results. Analysis of intra-model performance reveals that, for five of the seven architectures tested, larger patch sizes correspond to higher performance on the validation set. Regarding the validation metric value, there is an average difference of 2.6 points when comparing the performance of the larger patch size to the intermediate patch size and up to 5.5 points for nnUNet. Nevertheless, for two of the seven architectures (namely UNETR and VT-UNet), a gain in performance is observed with an intermediate patch size. For all architectures except nnFormer, the small patch size results in lower performance than the intermediate and large configurations.

**Table 5:** Performance per model over validation metric value (VMV) per patch size. Baselines appear in Italic and best-performing configurations in bold.

Model	VMV		
	Small	Intermediate	Large
nnFormer	64.2	*64.1*	**70.4**
nnUNet	63.1	63.5	***69.0***
SegmentationNet	50.3	*60.2*	**63.6**
Swin-UNETR	52.3	59.8	***60.4***
TransBTS	44.4	*49.6*	**56.1**
UNETR	36.3	***39.4***	37.1
VT-UNet	54.8	***58.1***	56.4
Average	52.2 ± 9.2	56.4 ± 8.2	**59.0 **±** 10.3**

**Figure 5:**
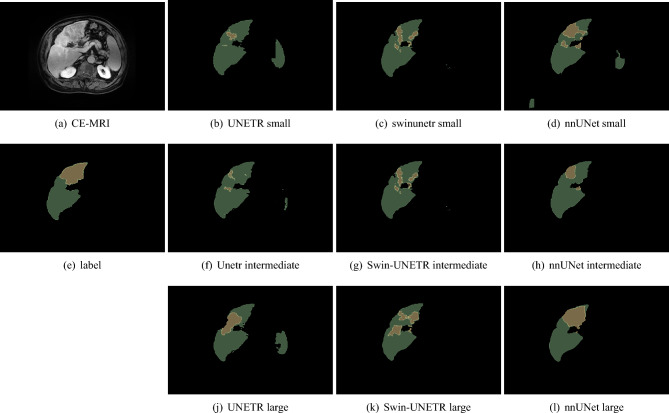
Illustration of the impact of the patch size on the segmentation performance for three different models: UNETR, Swin-UNETR and nnUNet.

Looking at Fig. 5, it can be observed that smaller patch sizes lead to a significantly higher number of artefacts, even for the best performing architectures. In particular, most segmentation models tend to misclassify the spleen as the liver when using small patch sizes. Larger patches lead to an improvement in the delineation of liver and liver tumour. Nevertheless, even with a large patch size, low-performing models encounter difficulty in eliminating all artefacts.

### Pre-processing and data augmentation influence

The effects of optimising the pre-processing and data augmentation for each model on the validation set are summarised in Table 7 with the best configuration per architecture in Table 6. The detailed results on the test set are given in Online Appendices Tables S3 and S4.

**Table 6:** Selected data pre-processing and data augmentation configuration per architecture with normalisation (norm), image fidelity (IF), scaling and resolution (SC) and luminance and contrast (LC) groups.

Model	Norm	IF	SR	LC
nnFormer	Range/std	✓	✓	✓
nnUNet	Min/max	✗	✗	✓
SegmentationNet	Min/max	✓	✗	✗
Swin-UNetr	Min/max	✗	✓	✓
Transbts	Min/max	✗	✓	✓
UNetr	Range/std	✗	✓	✓
VT-UNet	Range/std	✗	✗	✗

In terms of validation metric value, this optimisation leads to an average gain of 2.5 points when compared to the previous phase. Specifically, TransBTS and UNETR models experienced gains above four points. Nevertheless, a decrease in performance of 1.3 points can be observed for the nnFormer architecture.

**Table 7:** New validation metric value (VMV) obtained during the pre-processing and data augmentation optimisation compared to the initial VMV obtained after the patch size optimisation.

Model	Initial VMV	New VMV	Difference
nnFormer	**70.4**	69.1	−1.3
nnUNet	69.0	**69.7**	+0.7
SegmentationNet	63.6	**66.3**	+3.3
Swin-UNETR	60.4	**63.2**	+2.8
TransBTS	56.1	**60.4**	+4.3
UNETR	39.4	**43.8**	+4.4
VT-UNet	58.1	**61.1**	+3.0
Average	59.6 ± 9.6	**61.9 **±** 8.1**	+2.5

**Figure 6:**
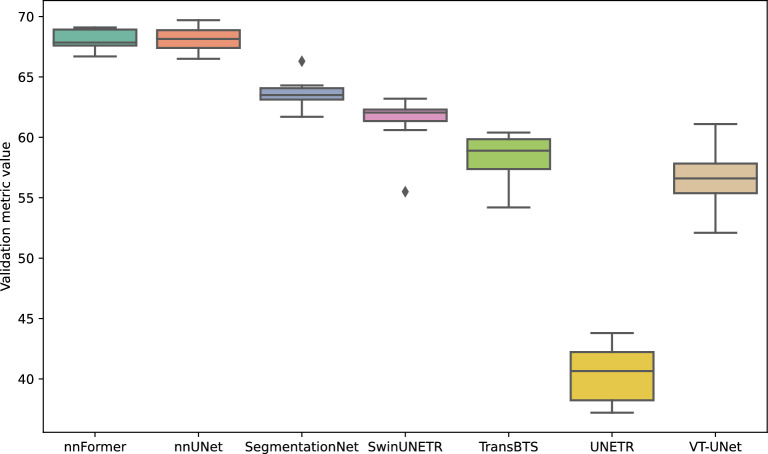
Performance variability across the validation metric value for seven deep learning architectures during the pre-processing and data augmentation optimisation phase.

Regarding the impact of each parameter across all tested combinations on the validation metric value, min–max normalisation results in an average gain of 2.3 points as compared to the standard-score normalisation. Incorporating scaling and resolution augmentations led to an average gain of 1.7 points and the image fidelity augmentation in an average gain of 1.9. However, the implementation of luminance and contrast augmentations caused a loss of 2.9 points. Figure 6 shows the variation of the performance of each tested combination per architecture during this phase.

### Learning paradigm influence

The effects of optimising the learning paradigm for each model are summarised in Table 9 and the best configuration in Table 8. Detailed results on the test set can be found in Online Appendices Tables S5 and S6.

**Table 8:** Selected learning paradigm configuration per architecture.

Model	Loss	Optimiser	Include background
nnFormer	DiceCE	SGD	✗
nnUNet	DiceCE	Adam	✗
SegmentationNet	DiceCE	Adam	✓
Swin-UNetr	DiceCE	AdamW	✓
Transbts	Dice	SGD	✗
UNetr	Dice	Adam	✗
VT-UNet	DiceCE	AdamW	✗

In terms of the validation metric value, this optimisation results in an average gain of 0.6 points in comparison to the previous phase. However, for four out of the seven architectures, there is a reduction in performance up to 2.6 points for VT-UNet. Nevertheless, this phase results in a gain of 6.0 points for TransBTS.

**Table 9:** Final validation metric value (VMV) obtained after the learning paradigm optimisation phase compared to the initial VMV obtained after the pre-processing and data augmentation phase.

Model	Initial VMV	Final VMV	Difference
nnFormer	**70.4**	69.7	−0.7
nnUNet	69.7	**70.9**	+1.2
SegmentationNet	**66.3**	65.2	−1.1
Swin-UNETR	**63.2**	62.8	−0.4
TransBTS	60.4	**66.4**	+6.0
UNETR	43.8	**45.8**	+2.0
VT-UNet	**61.1**	58.5	−2.6
Average	62.1 ± 8.3	62.8 ± 7.9	+0.6

**Figure 7:**
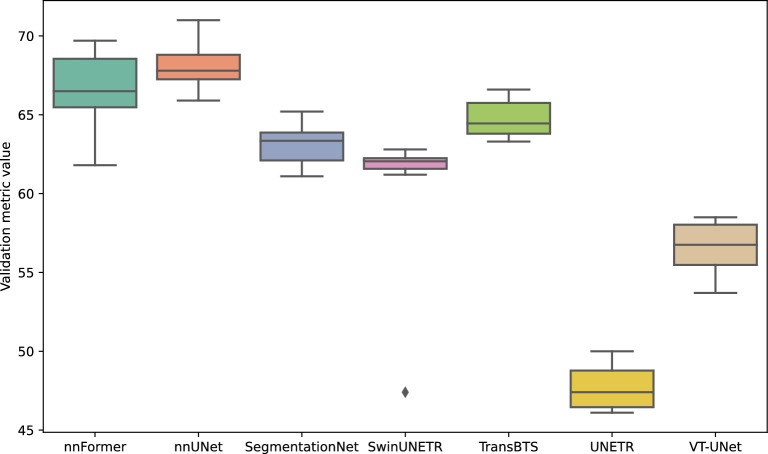
Performance variability across the validation metric value for seven deep learning architectures during the learning paradigm optimisation phase.

Looking at the average of all the parameter combinations studied during this phase, the use of the Adam optimiser led to an average improvement of 1.4 points over AdamW and 2.6 points over SGD based on the validation metric value. Notably, the DiceCE loss outperformed the Dice loss with an average gain of 0.9 points. On the other hand, the choice to incorporate the background in the loss calculation appears to have an insignificant impact on the models’ performance, with an average difference of only 0.2 points supporting its inclusion. Figure 7 shows the variation of the performance of each tested combination per architecture during this phase.

### Overall progression on the test set

Upon optimising the models, we identified the best hyperparameter sets for each. We then evaluated their performance on the test set. The following sections detail how these models fared in liver and tumour segmentation tasks, offering an analysis of the chosen parameters’ efficacy.

In Table 10, we present the progression of the performance throughout the entire optimisation process when testing on the liver dataset. The table highlights the differences between the initial baseline results and the highest performance achieved during training, based on the validation criteria. Improvements can be observed across all models leading to significant improvement for most of the calculated metrics, with an average increase of 1.7% in the Dice coefficient and a reduction of 36.7 mm in the Hausdorff distance. Nevertheless, the improvements in the nnUNet model - the leading baseline for this task - are marginal. Despite varying baseline performances, the optimisation process levelled the playing field, with all seven models achieving Dice coefficients within a narrow range of 92.3–95.1%. Although initially different, the baselines’ performances became similar after the optimisation process, as indicated by all seven models presenting a Dice coefficient between 92.3% and 95.1%.

**Table 10:** Performance evolution per model on the test dataset, for the liver between the baseline (BS) and the optimised hyperparameter combination (OP).

Model	Dice			5 mm SD			Precision			Recall			HD (mm)		
	BS	OP	*p*	BS	OP	*p*	BS	OP	*p*	BS	OP	*p*	BS	OP	*p*
nnFormer	94.2	**95.0**	**	91.9	**94.5**	***	94.6	**95.6**	*	93.9	**94.5**	_	72.9	**48.8**	***
nnUNet	***95.1***	95.0	_	94.6	**95.0**	_	94.7	**94.9**	_	***95.4***	95.1	_	**38.8**	39.0	_
SegmentationNet	**94.6**	94.3	_	**93.9**	93.7	_	94.8	**95.0**	_	**94.5**	93.7	**	52.0	**30.0**	*
Swin-UNETR	89.6	**94.4**	***	82.9	**93.1**	***	85.0	***96.4***	***	***95.4***	92.5	***	131.5	**42.5**	***
TransBTS	90.5	***95.1***	***	86.7	***95.1***	***	88.9	**95.9**	***	92.8	**94.4**	***	134.0	***24.9***	***
UNETR	91.9	**92.3**	–	86.8	**87.7**	_	91.3	**93.1**	***	**92.9**	91.9	***	**104.8**	117.3	_
VT-UNet	92.8	**94.4**	**	89.1	**93.3**	***	91.4	**94.6**	***	**94.6**	94.3	_	86.1	**41.0**	_

Italic indicates the best inter-model value for each metric. Stars indicate the level of significance of differences between baseline and optimised results based on paired t-test and Wilcoxon signed-rank test depending on the distribution of the results on the test set according to the Shapiro-Wilk test (no star means not significant, * means *p* < 0.05, ** means *p* < 0.01, and *** means *p* < 0.001).

**Figure 8:**
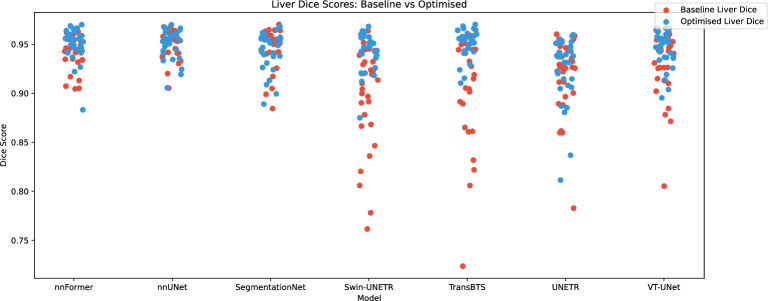
Comparison of the dice performance on liver segmentation of the models across the 30 images of the test set for the seven deep learning architectures between the baseline models and the optimised models.

Figure 8 offers a visual representation of the evolution of the performances on the test set between baseline (in red) and optimised (in blue) models on the liver segmentation task. This figure clearly highlights the positive impact of the optimisation process in particular for initially low-performing models. For TransBTS and Swin-UNETR for instance, before optimisation, respectively 12 and 14 images have a dice score below 90% against only one after optimisation.

Table 11 details the progression of performance achieved through the optimisation process on the tumour segmentation task of the test set. The optimisation process enhanced every model’s performance, demonstrated through higher Dice coefficient and Hausdorff distance scores. On average, the models gained an increase of 4.8% and 31mm in these metrics after the optimisation process. On low-performing models, this is traduced by a significant improvement in performance according to the paired t-test / Wilcoxon signed-rank test on the majority of the metrics. High-performing models such as nnUNet and nnFormer still show a gain in performance. They recorded Dice coefficients of 68.1% and 69.7%, respectively, making them the clear front runners by a considerable margin.

**Table 11:** Performance evolution on the test dataset, for the tumour between the baseline (BS) and the optimised hyperparameter combination.

Model	Dice			5mm SD			Precision			Recall			HD (mm)		
	BS	OP	p	BS	OP	p	BS	OP	p	BS	OP	p	BS	OP	p
nnFormer	68.5	***69.7***	_	67.6	**67.7**	_	78.9	**82.9**	_	**65.7**	64.4	_	**66.7**	72.3	_
nnUNet	**68.1**	**68.1**	_	67.5	***67.8***	_	78.8	***84.2***	_	***67.9***	63.9	_	60.2	**60.1**	_
SegmentationNet	55.8	**61.7**	_	52.5	**58.3**	_	61.6	**74.2**	_	55.1	**59.2**	_	91.5	**84.8**	_
Swin-UNETR	47.7	**55.1**	_	42.0	**50.9**	***	48.7	**78.2**	*	**57.2**	48.0	***	162.9	***58.5***	**
TransBTS	51.4	**62.1**	**	46.2	**59.2**	_	58.4	**73.1**	***	53.6	**59.9**	*	176.7	**73.7**	***
UNETR	33.1	**41.5**	***	30.0	**35.3**	*	40.9	**50.7**	_	31.9	**39.2**	**	**121.7**	138.1	*
VT-UNet	54.3	**56.6**	_	49.6	**53.9**	_	61.1	**71.6**	_	55.8	**56.0**	**	98.0	**72.7**	_

Italic indicates the best inter-model value for each metric. Stars indicate the level of significance of differences between baseline and optimised results based on paired t-test and Wilcoxon signed-rank test depending on the distribution of the results on the test set according to the Shapiro-Wilk test (no star means not significant, * means *p* < 0.05, ** means *p* < 0.01, and *** means *p* < 0.001).

**Figure 9:**
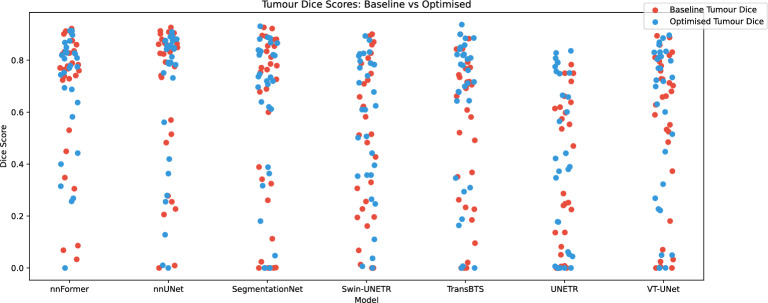
Comparison of the dice performance on tumour segmentation of the models across the 30 images of the test set for the seven deep learning architectures between the baseline models and the optimised models.

Figure 9 offers a visual representation of the evolution of the performances on the test set between the baseline (in red) and the optimised models (in blue) on the tumour segmentation task. In contrast to the liver segmentation task, the performance per image presents a high variability from one to another ranging from 0% to 95% of Dice.

## Discussion

### Impact of the different hyperparameters

In this study, a set of hyperparameters has been optimised through Bayesian search for seven distinct deep learning segmentation architectures. The hyperparameters were categorised into three sections: patch size, pre-processing and data augmentation, as well as learning paradigms.

#### Patch size influence

It is worth noting that traditional CNNs typically allows larger image inputs compared to transformer-based architectures. Thus, with CNN, we were able to load a large part of the images which provide significant context to the network. In contrast, transformers face difficulty to load images with patch sizes larger than $224 \times 224 \times 64$ pixels. Tests on the influence of patch size have revealed that a larger patch size results in a considerable enhancement in overall performance. In medical imaging, a significant context is vital in achieving optimal results. As a consequence, there is a need for more substantial GPUs. However, depending on the training architecture, training models with larger patch sizes for convergence has taken up to twice as much time compared to models with intermediate patch sizes.

#### Pre-processing and Data Augmentation influence

As illustrated in Fig. 6, the effects of pre-processing and data augmentation appear to rely heavily on the inherent performance capabilities of the particular deep learning model. The resilience of high-performing models like nnFormer and nnUNet is remarkable, as evidenced by their validation metric’s relatively low standard deviations. Architectures with lower inherent performance, such as UNETR and VT-UNet, show a significant sensitivity to these strategies. Consequently, customised optimisation methods that are tailored for specific deep learning architectures and contingent on their baseline performance become crucial.

#### Learning Paradigm influence

In contrast to data augmentation, the influence of the learning paradigm on the baseline performance of the model is not striking, as demonstrated in 7. When examining nnFormer and nnUNet, both models showed a greater sensitivity to these parameters. In contrast, models with lower baseline scores, namely SegmentationNet and TransBTS, exhibited comparatively stable results, potentially possibly indicating less influence of the learning paradigm or saturation of their performance capabilities within the current parameters.

The nuances observed highlight the complexity involved in optimising deep learning architectures. Selecting an appropriate learning paradigm is not simply a matter of best fitting the available data, but requires consideration of the unique characteristics and inherent capabilities of each architecture. A single strategy may not be sufficient, adapting it to the architecture can, in some cases, lead to notable performance improvements.

### Overall optimisation impact

Although almost all the model present progress over the different metrics on the liver and tumour segmentation according to Tables 10 and 11, the analysis of Figs. 8 and 9 exposes a different pattern in the impact of the optimisation on the two tasks.

In the context of liver segmentation, the optimisation process not only minimises the number of outliers in lower-performing models but also offers a noticeable improvement in performance, even for images that were already well segmented. Regarding tumour segmentation, most models show a modest improvement for each image analysed. However, it is important to note that images that initially demonstrated low performance prior to optimisation rarely show a significant increase in performance after optimisation. Therefore, these cases can still be classified as outliers.

On an inter-model point of view, after optimisation, if the performance on the liver is similar between the tested models, the performance on tumour is highly variable. Looking at Fig. 9, the images of the test set can be separated into two distinct categories based on performance: those with dice exceeding 60% and those falling below this threshold. While the models tend to exhibit comparable performances in the former group, it is in the latter category where performance disparities become more pronounced. This variance can be attributed to the ability of the best-performing models to excel in handling more complex cases, thus outperforming their average counterparts.

### CNN, transformer and hybrid models comparison

This study conducts a categorical comparison between CNNs, transformers, and hybrid models, revealing some noteworthy insights. It is evident that, following optimisation, hybrid networks not only compete with CNNs but can also surpass them, despite having a significantly smaller patch size. Additionally, advancements in GPU technology, particularly in terms of enhanced memory capacity, allowing for larger patch size could further cement hybrid networks as the predominant model in the field. Since VT-UNeT stands as the sole purely transformer-based network in this study, making definitive conclusions is challenging. However, the current data suggests that convolution-free models may not yet be a completely reliable alternative.

### Literature comparison

In the field of liver and liver tumour segmentation, there is a dearth of studies providing results on mono-modal MRI. Comparisons with other datasets are not consistently applicable due to distinctive imaging protocols and patient groups. Nevertheless, the work by Christ et al.^35^, as the only study conducted within a similar imaging modality (diffusion weighted MRI), remains relevant. They reported a Dice coefficient of 87% for liver and 69.7% for tumour segmentation across 31 patients. On the liver, the CHAOS segmentation challenge^66^ saw a dice score of 95.2%.

### Bayesian search time requirements

The specifics regarding the number of parameters, training duration, and inference time for the optimised configurations are meticulously outlined in Table 12. Each model exhibits an acceptable inference time per image from a clinical perspective, ranging between 0.6 and 3.0 s. However, there is considerable variability in training times, spanning from 17 to 144 h. This wide range could present challenges in the context of Bayesian optimisation.

Although Bayesian search offers an elegant strategy for fine-tuning hyperparameters, it has inherent limitations when it comes to training models in parallel. Unlike grid or random search methods, which can train numerous hyperparameter combinations simultaneously, the Bayesian approach works sequentially while determining the next evaluation based on the findings of previous ones. In our research context, evaluation necessitated testing 19 unique combinations. Due to the maximum training duration of 6 days per combination, the overall process lasted approximately 16 weeks. This restriction poses obstacles especially when dealing with complicated models or extensive datasets, and can markedly extend the tuning phase.

**Table 12:** Number of parameters, training time and inference time per model after optimisation.

Model	nb parameters	Training time (h)	Inference time per image (s)
nnFormer^43^	$3.7 \times 10^{7}$	144	1.7
nnUNet^33^	$3.0 \times 10^{7}$	121	0.8
SegmentationNet^44^	$1.8 \times 10^{7}$	28	0.6
Swin-UNetr^45,46^	$6.2 \times 10^{7}$	77	2.4
Transbts^48^	$3.4 \times 10^{7}$	69	1.1
UNetr^49^	$9.3 \times 10^{7}$	17	0.7
VT-UNet^50^	$1.2 \times 10^{7}$	77	3.0

### Limitations

The primary objective of this study was to identify the best hyperparameter configuration without utilising external data. However, due to the need to limit the computational expenses, several hyperparameters were intentionally not examined in this study. Here are the limitations of our study:

- **Image generation techniques:** hyperparameters that were not examined in this study involve generating images for data augmentation and the effect of pre-training or self-supervised learning. Although a self-supervised version of SWIN-UNETR was applied, its impact on performance was not assessed. According to Shin et al.^67^, if image generation is achievable without external data, its impact is negligible when other sources of data augmentation are employed
- **Mono-centre dataset:** all images in the ATLAS dataset were obtained from patients at the University Hospital of Dijon, France. As a result, conclusions reached in this study may vary with a more diverse dataset.
- **Cross-validation omission:** cross-validation was not utilised in our study due to the extended training period of the deep learning architectures examined. This choice could influence the reliability of our performance metrics and their applicability to diverse data samples.
- **Loss functions:** our focus was limited to the techniques employed in the seven tested pipelines. In particular, we did not examined loss functions customised for specific assignments such as tumour segmentation. These encompassed the Dice topK loss, the boundary loss, and the Hausdorff distance loss.
- **Architecture modifications:** our study refrained from altering the original architectures in relation to depth, width or layer order. The majority of these architectures were developed with datasets of different complexities in mind. Consequently, deviations to these architectures could result in diverse outcomes, particularly when applied to datasets of simpler or more complex natures than originally anticipated.

## Conclusion

Our study emphasises the significance of hyperparameter optimisation in the field of medical imaging, particularly in the 3D segmentation of the liver and liver tumour on CE-MRI. By implementing Bayesian search on a subset of hyperparameters, it was possible to clearly measure the effects of patch size, pre-processing, data augmentation and learning paradigms, whilst still keeping the quantity of tested combinations reasonable. An average gain per architecture of 1.7% for the liver and a remarkable 5.0% for the liver tumour using the Dice coefficient illustrates the significance of hyperparameter tuning. Indeed, on such complex tasks, only a few methodological suggestions have resulted in analogous progressions in segmentation quality. However, although among the tested architectures all of them appear to be effective in the literature, it is important to note that significant performance discrepancies have been observed between them, which does not depends on the hyperparameter configurations. Nevertheless, hybrid transformer architectures, typically associated with large datasets, have shown their capability to match CNN performance even with limited data. Thus, for future directions, it appears promising to expand the dataset size through image generation, self-supervised and mixed supervised learning. This is especially true given the potential of transformers to benefit from such expansions. This study encourages a re-evaluation of the role of transformers in scenarios with limited data and highlights their emerging relevance in medical image segmentation. Such an improvement in automatic segmentation represents a significant step towards the potential automation of this process, reducing the reliance on manual segmentation performed by radiologists. This advancement not only streamlines the workflow but also promises to improve patient care by ensuring more precise and timely diagnoses and SIRT treatment planning.

### Supplementary Information

Supplementary Tables.

## Data Availability

The data that support the findings of this study are publicly available at https://atlas-challenge.u-bourgogne.fr.
