# Supplementary material for: Navigating the nuances: comparative analysis and hyperparameter optimisation of neural architectures on contrast-enhanced MRI for liver and liver tumour segmentation
Source: Sci Rep. 2024 Feb 12;14:3522. doi: 10.1038/s41598-024-53528-9 (PMC10861452; doi:10.1038/s41598-024-53528-9)
Supplement: Supplementary file 1 — Supplementary Tables. [file 41598_2024_53528_MOESM1_ESM.pdf]

Appendix

0.1 Patch size optimisation

Table S1. Performance evolution on test images over liver during the patch size optimisation. BS corresponds to values obtained by the baseline model and PS by the retained models at the end of the patch size optimisation. Dash symbols correspond to the models for which the baseline corresponds to the retained configuration.

Model	Dice		5mm SD		Precision		Recall		HD (mm)	
	BS	PS	BS	PS	BS	PS	BS	PS	BS	PS
nnFormer	94.2	95.0	91.9	94.5	94.6	95.6	93.9	94.5	72.9	48.8
nnUNet	–	95.1	–	94.6	–	94.7	–	95.4	–	38.8
SegmentationNet	94.6	94.3	93.9	93.7	94.8	95.0	94.5	93.8	52.0	46.4
Swin-UNETR	–	89.6	–	82.9	–	85.0	–	95.4	–	131.5
TransBTS	90.5	93.7	86.7	92.2	88.9	97.4	92.8	90.3	134.0	52.0
UNETR	–	91.9	–	86.8	–	91.3	–	92.9	–	104.8
VT-UNet	–	92.8	–	89.1	–	91.4	–	94.6	–	86.1

Table S2. Performance evolution on test images over tumour during the patch size optimisation. BS corresponds to values obtained by the baseline model and PS by the retained models at the end of the patch size optimisation. Missing values corresponds to the models for which the baseline corresponds to the retained configuration.

Model	Dice		5mm SD		Precision		Recall		HD (mm)	
	BS	PS	BS	PS	BS	PS	BS	PS	BS	PS
nnFormer	68.5	69.7	67.6	67.7	78.9	82.9	65.7	64.4	66.7	72.3
nnUNet	–	68.1	–	67.5	–	78.8	–	67.9	–	60.2
SegmentationNet	55.9	57.7	52.5	53.8	61.6	67.9	55.1	56.5	91.5	85.4
Swin-UNETR	–	47.7	–	42.0	–	48.7	–	57.2	–	162.9
TransBTS	51.4	55.5	47.9	50.3	60.4	74.1	53.6	48.7	171.5	88.8
UNETR	–	33.1	–	30.0	–	40.9	–	31.9	–	121.7
VT-UNet	–	54.3	–	49.6	–	61.1	–	55.8	–	98.0

0.2 Pre-processing and data augmentation optimisation

Table S3. Performance evolution on test images over liver during the data pre-processing and data augmentation optimisation. PS corresponds to values obtained by the patch size optimised models and DA by the retained models at the end of the data pre-processing and data augmentation optimisation.

Model	Dice		5mm SD		Precision		Recall		HD (mm)	
	PS	DA	PS	DA	PS	DA	PS	DA	PS	DA
nnFormer	95.0	95.0	94.5	94.7	95.6	95.8	94.5	94.3	48.8	27.8
nnUNet	95.1	95.1	94.6	95.2	94.7	95.3	95.4	94.9	38.8	27.0
SegmentationNet	94.3	94.3	93.7	93.7	95.0	95.0	93.8	93.7	46.4	30.0
Swin-UNETR	89.6	94.4	82.9	93.1	85.0	96.4	95.4	92.5	131.5	42.5
TransBTS	93.7	94.4	92.2	93.2	97.4	95.8	90.3	93.1	52.0	45.2
UNETR	91.9	93.1	86.8	90.2	91.3	94.3	92.9	92.1	104.8	62.7
VT-UNet	92.8	94.4	89.1	93.3	91.4	94.6	94.6	94.3	86.1	41.0

Table S4. Performance evolution on test images over tumour during the data pre-processing and data augmentation optimisation. PS corresponds to values obtained by the patch size optimised models and DA by the retained models at the end of the data pre-processing and data augmentation optimisation.

Model	Dice		5mm SD		Precision		Recall		HD (mm)	
	PS	DA	PS	DA	PS	DA	PS	DA	PS	DA
nnFormer	69.7	66.1	67.7	65.5	82.9	76.2	64.4	63.7	72.3	53.1
nnUNet	68.1	68.0	67.5	66.4	78.8	73.1	67.9	71.7	60.2	70.0
SegmentationNet	57.7	61.7	53.8	58.3	67.9	74.2	56.5	59.2	85.4	84.8
Swin-UNETR	47.7	55.1	42.0	50.9	48.7	78.2	57.2	48.0	162.9	58.5
TransBTS	55.5	55.7	50.3	51.4	74.1	74.6	48.7	49.1	88.8	86.8
UNETR	33.1	43.1	30.0	36.5	40.9	49.6	31.9	41.8	121.7	118.7
VT-UNet	54.3	56.6	49.6	53.9	61.1	71.6	55.8	56.0	98.0	72.7

0.3 Learning paradigm optimsation

Table S5. Performance evolution on test images over tumour during the learning paradigm optimisation. DA corresponds to values obtained by the retained models at the end of the data pre-processing and data augmentation optimisation and LP by the retained models at the end of the learning paradigm optimisation.

Model	Dice		5mm SD		Precision		Recall		HD (mm)	
	DA	LP	DA	LP	DA	LP	DA	LP	DA	LP
nnFormer	95.0	94.7	94.5	94.7	95.6	95.6	94.5	93.8	48.8	33.5
nnUNet	95.1	95.0	95.2	95.0	95.3	94.9	94.9	95.1	27.0	39.0
SegmentationNet	94.3	91.6	93.7	89.2	95.0	958	93.7	88.7	30.0	41.8
Swin-UNETR	94.4	94.9	93.1	94.4	96.4	94.8	92.5	91.1	42.5	47.3
TransBTS	94.4	95.1	93.2	95.1	95.8	95.9	93.1	94.4	45.2	24.9
UNETR	93.1	92.3	90.2	87.7	94.3	93.1	92.1	91.9	62.7	117.3
VT-UNet	94.4	93.8	93.3	91.3	94.6	93.9	94.3	93.8	41.0	69.9

Table S6. Performance evolution on the test dataset, for the tumour during the learning paradigm optimisation. DA corresponds to values obtained by the retained models at the end of the data pre-processing and data augmentation optimisation and LP by the retained models at the end of the learning paradigm optimisation.

Model	Dice		5mm SD		Precision		Recall		HD (mm)	
	DA	LP	DA	LP	DA	LP	DA	LP	DA	LP
nnFormer	69.7	68.2	67.7	67.4	82.9	83.5	64.4	61.1	72.3	68.2
nnUNet	68.0	68.1	66.4	67.8	73.1	84.2	71.7	63.9	70.0	60.1
SegmentationNet	61.7	58.3	58.3	54.3	74.2	70.8	59.2	58.6	84.8	85.8
Swin-UNETR	55.1	61.1	50.9	56.7	78.2	78.2	48.0	55.8	58.5	74.3
TransBTS	55.7	62.1	51.4	59.2	74.6	73.1	49.1	59.9	86.8	73.7
UNETR	43.1	41.5	30.0	35.3	49.6	50.7	41.8	39.2	118.7	138.1
VT-UNet	56.6	55.9	53.9	52.9	71.6	63.8	56.0	56.6	72.7	100.5
